# Challenging bone biopsies: what radiologists needs to know

**DOI:** 10.1007/s00256-026-05336-7

**Published:** 2026-08-10

**Authors:** Leonor Garbin Savarese, Nicolas Papalexis, Mateus de Andrade Hernandes, Paolo Spinnato, Giancarlo Facchini, Marco Miceli, Nelson Fabricio Gava, Edgard Eduard Engel, Marcello Henrique Nogueira-Barbosa

**Affiliations:** 1 Department of Medical Imaging, Hematology and Clinical Oncology, Ribeirao Preto Medical School, University of Sao Paulo, Av. Bandeirantes, 3900 CEP, Ribeirão Preto, São Paulo 14049-090 Brazil; 2 Department of Radiology, ARNAS G. Brotzu – Businco Oncologic Hospital, Cagliari, Italy; 3 Diagnostic and Interventional Radiology Unit, IRCCS Istituto Ortopedico Rizzoli, Bologna, Italy; 4 Department of Orthopedics and Anesthesiology, Ribeirao Preto Medical School, University of Sao Paulo, Ribeirão Preto, São Paulo Brazil

**Keywords:** Bone neoplasms, Bone diseases, Biopsy, Needle, Tomography, Radiology, Interventional

## Abstract

Bone biopsies play a pivotal role in the diagnostic workup of a wide range of bone pathologies. In many cases, image-guided percutaneous biopsy is favored over surgical approaches because it is minimally invasive, highly accurate, cost-efficient, and associated with lower morbidity. Among the available imaging modalities, CT guidance offers superior spatial resolution and enables precise needle placement, but certain skeletal locations remain technically demanding even under CT guidance. This review focuses on challenging CT-guided bone biopsy scenarios, including lesions adjacent to critical neurovascular or spinal structures, densely sclerotic lesions, and lesions occult on CT, and illustrates practical decision-making through representative clinical cases.

## Introduction

Image-guided percutaneous bone biopsy is a well-established, minimally invasive alternative to open surgical biopsy and plays a central role in the diagnostic workup of musculoskeletal lesions. Reported diagnostic accuracy ranges from approximately 68% to 99%, depending on lesion type, location, and biopsy technique [1–6]. CT guidance remains the main imaging modality for most bone biopsies because it provides excellent spatial resolution, precise needle positioning, and safe access to deep or surgically challenging lesions. In addition, percutaneous biopsy has become increasingly important for molecular profiling and personalized oncologic treatment strategies [7–9].

Although many lesions can be safely approached using standard techniques, some biopsies remain technically demanding because of lesion location, density, or limited conspicuity on CT. These include lesions adjacent to critical structures, densely sclerotic lesions, and CT-occult lesions requiring multimodality imaging correlation for accurate targeting. In these situations, careful preprocedural planning, detailed anatomical knowledge, and tailored biopsy strategies are essential to maximize diagnostic yield and minimize complications [2–4, 7, 8, 10–19].

This review discusses practical considerations and representative cases involving (1) lesions adjacent to critical structures, (2) densely sclerotic lesions, and (3) CT-occult lesions. Potential complications and key planning strategies are summarized in Table 1.

**Table 1:** Decision-making framework for challenging CT-guided bone biopsy scenarios

Challenging scenario	Primary planning concern	Key technical strategy	Biopsy system selection
Lesions adjacent to critical neurovascular structures	Risk of neural or vascular injury	Careful trajectory planning using long or alternative intraosseous access routes	Coaxial core biopsy systems (e.g., Bonopty-type systems)
Densely sclerotic lesions	Difficulty in cortical penetration and sampling	Use of powered biopsy systems and short core sampling	Battery-powered drill-assisted systems (e.g., OnControl-type systems)
CT-occult lesions	Inaccurate target localization	Correlation with MRI or PET/CT and landmark-based targeting	Standard coaxial core biopsy systems
Anatomically constrained locations	Limited margin for trajectory correction	Multiplanar planning and precise initial alignment	Rigid coaxial biopsy systems

## General technical considerations in unusual CT-guided bone biopsies

### Indications and decision-making: when should a bone lesion be biopsied?

Before planning a bone biopsy, the radiologist must first determine whether tissue sampling is truly indicated and likely to impact clinical management. Common indications include diagnosis of a primary bone tumor, confirmation of metastatic disease, evaluation of hematologic malignancies, and microbiologic or histopathologic diagnosis in suspected osteomyelitis or spondylodiscitis. In selected cases, biopsy may also be required for molecular profiling and treatment planning in oncologic patients [7–9].

This initial assessment should integrate clinical presentation, laboratory findings, and imaging characteristics. It is equally important to recognize lesions in which biopsy is generally not indicated (“no-touch lesions”), particularly when imaging findings are characteristic and the clinical scenario is appropriate. Examples include typical bone islands, non-ossifying fibromas, fibrous dysplasia, and classic vertebral hemangiomas. In these situations, unnecessary biopsy may expose the patient to avoidable risks and potentially misleading histopathologic findings.

It is crucial to ensure that an adequate imaging workup has been performed and that all available studies have been reviewed, including radiographs, CT, MRI, and nuclear medicine examinations. Correlation among imaging modalities helps refine the differential diagnosis, identify the most appropriate target when multiple lesions are present, and anticipate potential technical challenges [2–4, 7, 8, 10, 12, 13, 16]. In selected lesions with low clinical suspicion or benign imaging features, short-interval imaging follow-up may be a reasonable alternative to immediate biopsy.

## Preprocedural planning

### Imaging review and target selection

A structured preprocedural plan is essential, particularly for unusual bone lesions. Patient positioning should be considered because anatomical relationships and osseous landmarks may change between supine, prone, and lateral decubitus positions, especially in the pelvis and spine, affecting lesion conspicuity, trajectory, and adjacent critical structures. In suspected primary bone sarcoma, orthopedic oncology may request tattooing or marking of the biopsy tract to facilitate en bloc resection [2–4, 7, 8, 10, 12, 16]. CT remains the mainstay, but fluoroscopy, ultrasound, cone-beam CT, MRI, or PET/CT may be preferred or used adjunctively. Target selection should favor viable tissue and avoid necrotic, cystic, or hemorrhagic areas. Ultrasound can guide selected osteolytic lesions with cortical thinning or disruption or an extraosseous soft-tissue component, providing real-time needle visualization, Doppler assessment, and no ionizing radiation; high diagnostic yield has been reported in appropriately selected lesions [20]. Communication with the referring team is essential to confirm the diagnostic question [1, 4, 7, 8, 10, 12, 16, 18]. The workflow is summarized in Table 2.

**Table 2:** Workflow-oriented planning and technical considerations for CT-guided bone biopsy

Step	Key question	Practical considerations
Indication assessment	Is biopsy indicated and likely to impact management?	Consider clinical context and imaging features; recognize lesions with typical benign appearance where biopsy may not be indicated; consider short-interval imaging follow-up as an alternative in selected cases
Imaging review and target selection	Which lesion and which component should be sampled?	Review CT, MRI, and PET/CT; select the most representative, viable, or metabolically active portion; avoid necrotic or heavily mineralized areas when feasible
Trajectory planning	What is the safest access route?	Prefer the safest rather than the shortest trajectory; respect compartmental anatomy and potential surgical planes; consider long intraosseous trajectories in anatomically constrained regions
Anesthesia and sedation	How will patient discomfort be minimized?	Adequate local and periosteal anesthesia is essential; consider deep sedation or general anesthesia in prolonged procedures, painful lesions, or anatomically complex approaches
Biopsy system selection	Which biopsy system is most appropriate?	Coaxial systems are preferred in most cases; powered devices facilitate cortical penetration and sampling of sclerotic lesions; hand-held systems may be adequate in less sclerotic bone
Sampling strategy	How should tissue be obtained?	Obtain multiple representative cores rather than a single long specimen; consider FNA as a complementary technique in selected tumors; gentle suction may aid sampling in lytic lesions
Specimen handling	How should samples be managed after retrieval?	Coordinate specimen handling with pathology; formalin fixation for routine histology; fresh tissue submission in suspected hematologic malignancy; minimize crush artifact
Postprocedural management	How are adequacy and complications addressed?	Assess sample adequacy; manage hemostasis; consider tract management when indicated; monitor for complications

### Trajectory planning and compartmental considerations

When planning the biopsy trajectory, potential tract seeding should be considered in suspected primary bone tumors because the biopsy route may influence limb-sparing or reconstructive surgery. Safe access may require long intraosseous trajectories, such as trans-iliac or trans-pedicular approaches, to avoid neurovascular structures. The shortest path is not necessarily the safest, and accurate multiplanar planning is essential because trajectory correction becomes limited once the device is advanced within bone [3, 6, 10, 12, 21, 22].

### Anesthesia and sedation strategy

Adequate pain control is a critical component of preprocedural planning. Local anesthesia of the skin, subcutaneous tissues, and especially the periosteum is essential to reduce patient discomfort during cortical penetration [8, 23, 24]. In selected cases, such as long procedures, painful sclerotic lesions, pediatric patients, or anatomically constrained approaches (e.g., cervical spine or transoral access), deep sedation or general anesthesia may be considered in collaboration with anesthesiology [10].

#### Biopsy system selection

Coaxial biopsy systems are preferred in most cases [1, 4, 7, 8, 12, 16, 18]. An outer working cannula traverses the cortex and a removable trephine obtains multiple cores through a single cortical perforation. Maintaining the introducer permits controlled re-angulation or incremental advancement toward different lesion areas, cortical anesthetic injection, and stable needle positioning.

Needle gauge and length should be tailored to the lesion and body habitus. Bone biopsy systems typically range from 10–12 G to 14–16 G. Larger-bore needles provide more substantial cores for primary tumors or molecular testing, at the cost of slightly higher bleeding risk and firmer cortical penetration [2, 7, 8, 11, 12, 18]. Thinner needles may be advantageous near critical structures or in small bones, where minimizing cortical defects and fracture risk is important. Shorter needles provide better torque control in superficial lesions, whereas longer devices are required for deep pelvic, spinal, or sacral targets [4, 7, 10, 12, 13, 18]. Powered systems facilitate access to densely sclerotic lesions and reduce operator fatigue, but brief intermittent drilling and limited torque help reduce heat artifact, needle bending, and fracture [7, 8, 11, 12, 15, 17, 18, 25, 26]. Manual Jamshidi- or Bonopty-type systems remain suitable for lesions with limited sclerosis and when powered devices are unavailable or cost-prohibitive [15, 17, 27]. Spring-assisted or specimen-retaining mechanisms may improve capture of friable bone or marrow [22].

Soft-tissue core needles with cutting trays and spring-loaded mechanisms are useful for extraosseous components, which often yield more cellular material than mineralized bone [2, 7–9, 12, 18, 19, 22]. In very lytic lesions, they may also be advanced intraosseously through a defect created by the outer cannula, avoiding additional cortical trauma. Tip dead space should be considered when sampling small or soft lesions because it may reduce effective tissue capture. Combining a bone trephine for access with a soft-tissue core device allows sampling to be adapted to lesion consistency and anatomy.

#### Sampling strategy and specimen handling

Core biopsy, FNA, and specimen handling should be planned before the procedure. For suspected lymphoma, plasmacytoma, or other hematologic malignancies, close coordination with pathology is essential so that material is triaged for flow cytometry, immunophenotyping, and possible genomic studies [7, 8, 12, 16, 28]. In metastatic disease or primary tumors, tissue handling should preserve material for molecular profiling [8, 9, 18, 19, 29]. When feasible, a separate blood clot or soft-tissue sample should be submitted without decalcification to preserve nucleic acids for molecular and genetic analyses.

Core samples are generally fixed in formalin; in suspected hematologic malignancies, at least part of the specimen should be submitted fresh in an appropriate medium for ancillary studies. Gentle handling minimizes crush artifact, particularly in lymphoma [18, 30]. FNA may complement core biopsy in metastatic disease, plasma cell neoplasms, and selected hematologic malignancies, where cytology and ancillary studies can be informative.

#### Factors associated with improved diagnostic yield

Several technical and lesion-related factors influence diagnostic yield in bone biopsy, including lesion selection, sampling strategy, biopsy device, and specimen handling [7, 11, 12, 16–18, 29]. Larger-gauge needles, when safely applicable, may improve yield by providing more substantial tissue cores and facilitating ancillary studies such as immunohistochemistry and molecular profiling [2, 7, 11, 18, 29].

In osteolytic lesions, longer cores are generally desirable because they encompass a greater volume of viable tumor tissue. The “sandwich technique,” in which the biopsy specimen includes the lesion with adjacent normal bone on either side, may help preserve tissue architecture and improve diagnostic yield [28]. Gentle negative pressure applied during sampling may also facilitate tissue retrieval in friable lesions. In lesions with central necrosis, the most informative tissue is typically located at the enhancing peripheral zone and at the interface between viable tumor and bone [1, 12, 16, 18, 19, 22].

When performed coaxially through the same introducer, FNA provides additional cytologic material with minimal incremental risk [7, 8, 12, 16, 18, 22].

In densely sclerotic lesions, powered systems may facilitate cortical penetration, but less dense regions should be targeted whenever feasible to maximize diagnostic tissue [7, 8, 11, 12, 15, 17, 18, 25, 26, 32]. Detailed drilling strategies are discussed below.

Immediate on-site cytologic or adequacy assessment may improve diagnostic yield, particularly in hematologic malignancies, by confirming representative sampling and indicating whether additional cores are needed [31].

Intrinsic lesion-related factors also influence diagnostic success. Larger lesions, cortical destruction, extraosseous soft-tissue extension, and metabolically active tumors generally yield diagnostic samples more frequently [2, 7–9, 11, 12, 17–19, 29]. Conversely, small, purely intraosseous, minimally lytic, or low-grade lesions tend to have lower diagnostic yield and often require meticulous procedural planning.

An important exception is osseous lymphoma, which has been associated with lower diagnostic yield compared with metastatic disease, likely because of patchy marrow infiltration and frequent sclerosis [11]. In these cases, targeting the most lytic or metabolically active portion of the lesion and obtaining multiple representative cores may improve diagnostic performance. Close coordination with hematopathology is also essential, particularly when flow cytometry, immunophenotyping, or molecular studies are anticipated [7, 8, 12, 16, 18].

## Challenging scenarios

### Densely sclerotic and heavily mineralized lesions

Compared with lytic lesions, osteoblastic targets have lower diagnostic yield, with pooled rates around 74%. A subgroup analysis reported higher yield for battery-powered than manual drill systems (76.7% vs 65.2%) [7, 8, 11, 12, 15, 17, 18, 25, 29]. Technical challenges include difficult entry and slow advancement through compact bone, needle deflection or fracture, and core impaction within the trephine.

**Fig. 1:**
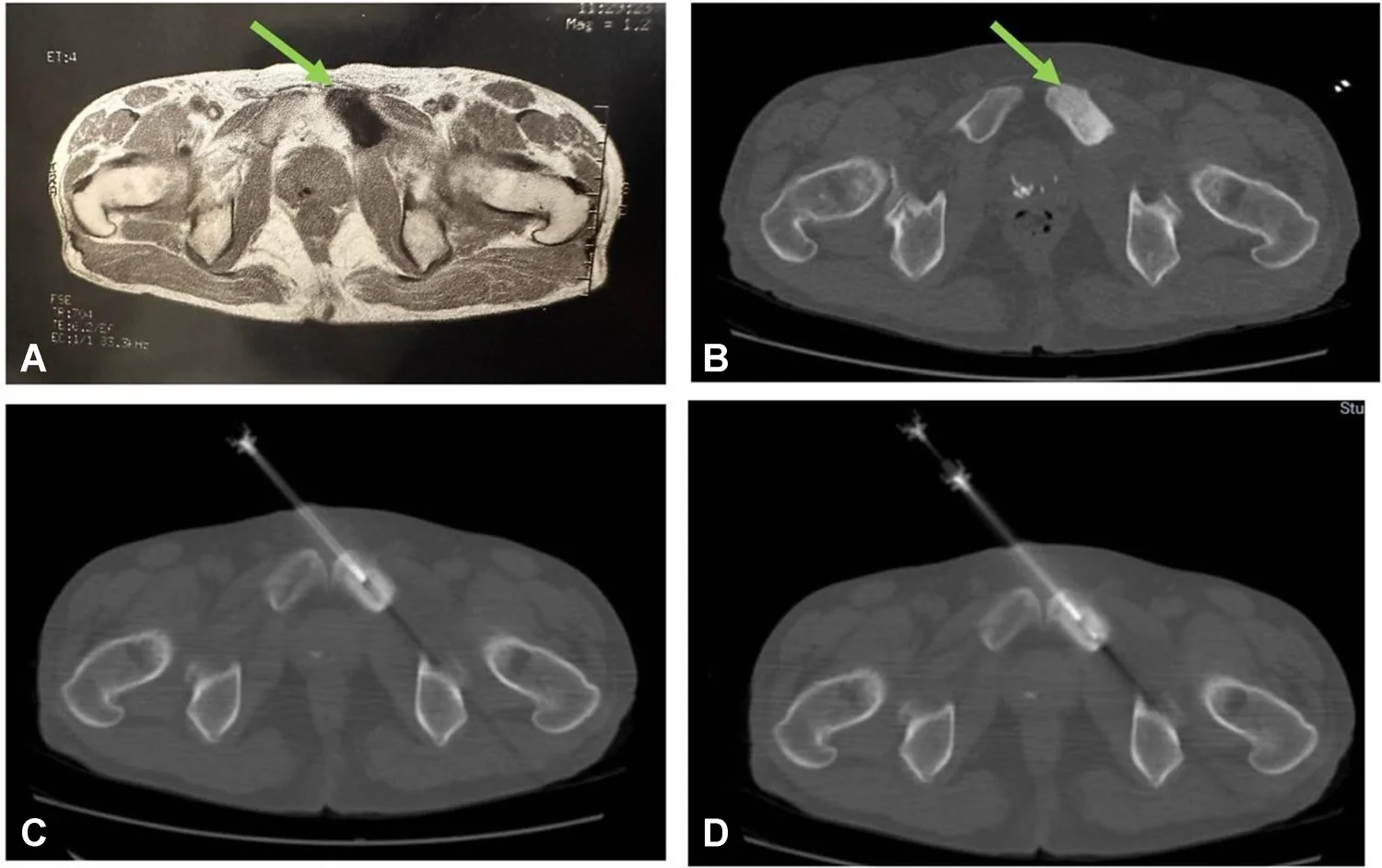
CT-guided biopsy of a densely sclerotic pubic bone lesion using a battery-powered drill. A 64-year-old man with a history of occult head and neck cancer presented with a densely sclerotic lesion in the left pubic bone (arrows in **A** and **B**). A CT-guided biopsy was performed using a coaxial bone biopsy system equipped with a battery-powered drill (**C**, **D**), which facilitated controlled cortical penetration and advancement through hard bone. Multiple short core specimens were obtained rather than a single long sample to minimize the risk of specimen impaction and needle blockage and to avoid excessive torque on the needle. This case illustrates key technical principles for approaching densely sclerotic lesions with drill-assisted short-core sampling

When using a battery-powered drill in sclerotic bone (Fig. 1), short cores (approximately 5 mm) are preferable to a single long cylinder because they reduce specimen impaction and allow multiple passes [15, 17]. If a core becomes lodged, a smaller-gauge trephine may be introduced coaxially to “biopsy the biopsy” and retrieve the material. Controlled tapping may also assist specimen release. Soaking the needle in hot water or a decalcifying solution is a last resort because it may impair histologic quality, although it can prevent complete sample loss [12, 16, 18].

Short-interval drilling with frequent pauses limits frictional heat and compressive forces, reducing crush artifact and frictional burn that may compromise histologic interpretation [15, 17, 25, 26]. Core length, drilling pattern, and rescue strategies for impacted tissue should therefore be adapted to the lesion.

### Lesions adjacent to critical neurovascular or spinal structures

Lesions in the craniovertebral junction, cervical spine, sacrum, ribs, sternum, and deep pelvis pose substantial technical and safety challenges [7, 10, 12–14, 18, 19, 21, 25]. The route should be planned with the referring surgeon, considering standard surgical approaches, compartmental anatomy, and preservation of future treatment options such as limb-sparing surgery or complex spinal reconstruction [3, 6, 10, 12, 22].

**Fig. 2:**
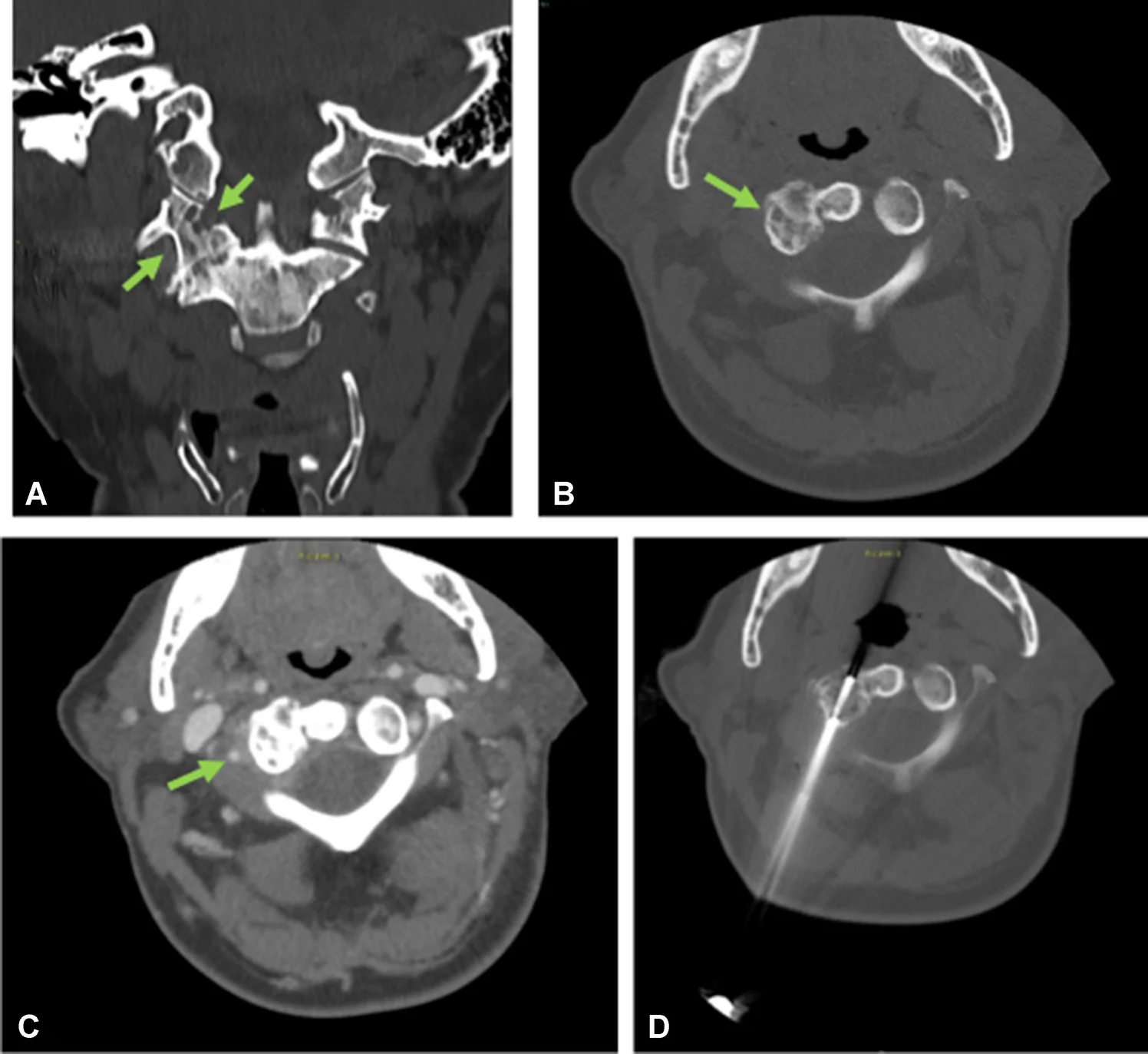
A 58-year-old woman with intense neck pain and reduced range of motion (head rotations). CT in the coronal (**A**) and axial (**B**) planes shows multiple bone erosions of the right C1–C2 articular processes involving both C1 and C2 lateral masses (arrows). Preliminary axial CT after contrast media injection (**C**) allows visualization of the vertebral artery (arrow) and planning of a safe posterior CT-guided biopsy approach (**D**). A final diagnosis of non-specific inflammatory-degenerative disease was achieved, excluding neoplasm or infection

**Fig. 3:**
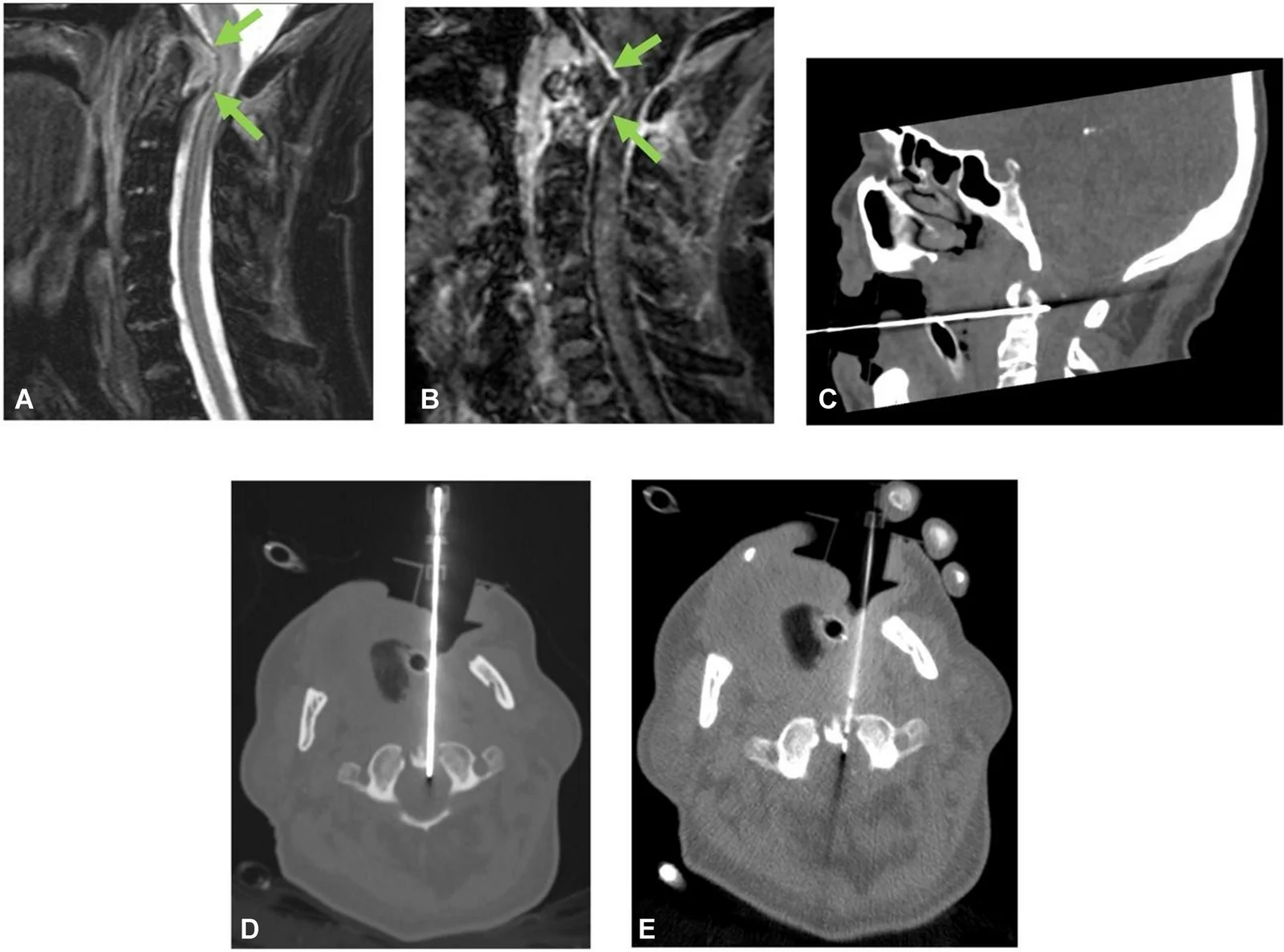
Transoral CT-guided biopsy of a retro-odontoid abscess in a 73-year-old man. The patient presented with cervical pain and restricted range of motion. Imaging demonstrated an inflammatory/infectious process involving the atlantoaxial joint, associated with a retro-odontoid abscess (arrows) (**A**, **B**). CT-guided transoral access was performed under general anesthesia. Initial aspiration of purulent material was obtained (**C**, **D**), followed by slight retraction of the needle toward the atlanto-odontoid joint. Through a coaxial system, a soft-tissue biopsy needle was then advanced for intra-articular tissue sampling (**E**). The endotracheal tube is partially visualized, confirming secured airway control during the intervention. This case illustrates how a transoral trajectory offers a short, direct path to retro-odontoid lesions while maintaining safe distances from critical neural and vascular structures. When combined with real-time CT fluoroscopy, this approach enables precise needle placement in a confined anatomical space, enhancing procedural safety and reducing overall procedure time

In the cervical spine, vertebral arteries, carotid sheath structures, or the spinal cord may preclude routine posterolateral (Fig. 2) or anterolateral approaches. A transoral route can provide direct access to selected retro-odontoid or C2 lesions, although working angles and maneuverability are limited (Fig. 3) [7, 10, 12–14, 18, 21]. Cross-sectional imaging, and CT angiography when needed, should define carotid and vertebral artery anatomy and variants that may influence the approach [1, 4, 7, 8, 10, 12–14, 18, 33].

**Fig. 4:**
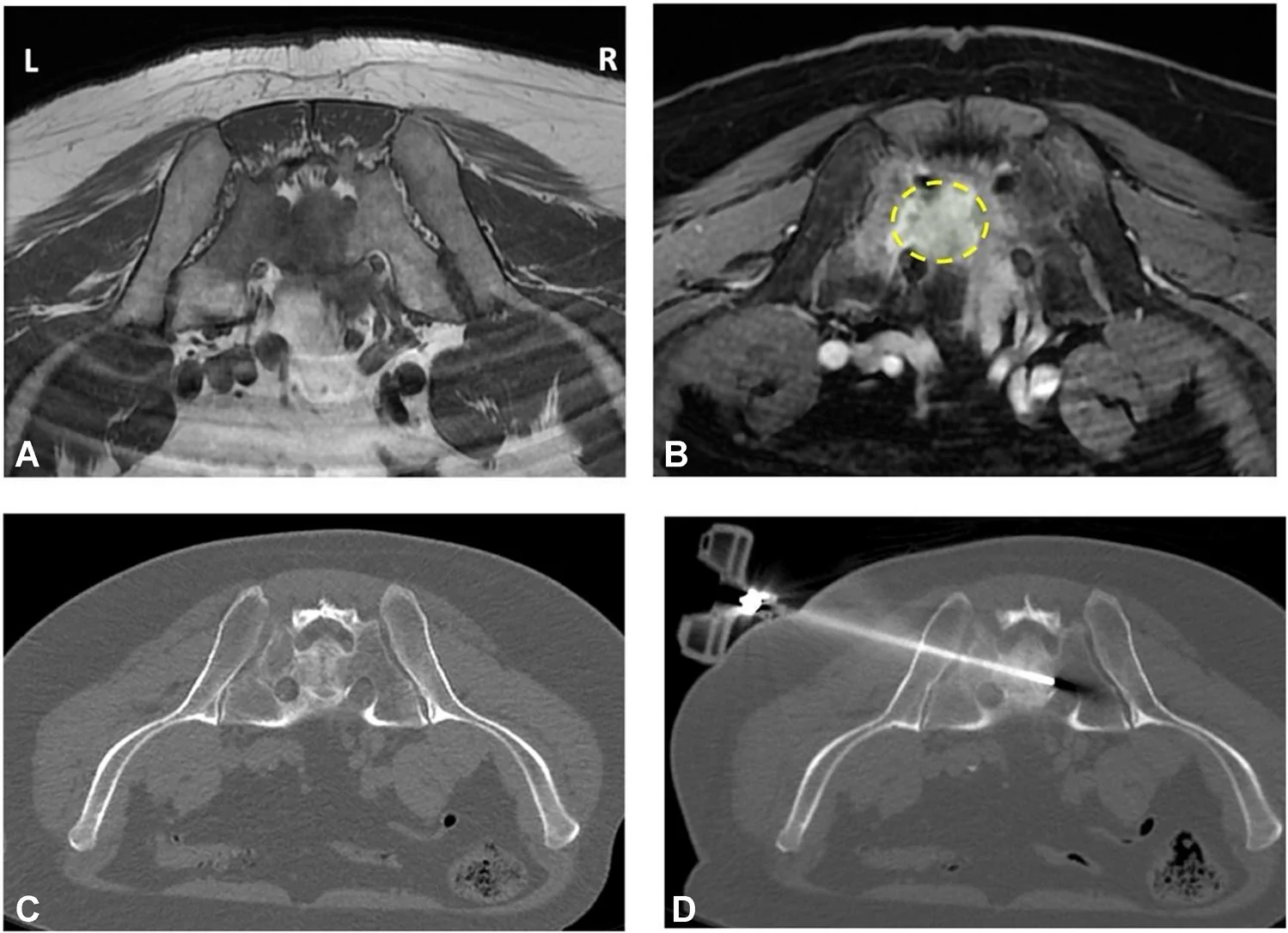
Long trans iliac trajectory for CT-guided biopsy of a sacral lesion. A 71-year-old man presented with sacral pain. Imaging identified a bone lesion in the sacrum. The patient had previously undergone an inconclusive biopsy of the sacral wing at an outside institution. Based on MRI findings, axial T2-weighted (**A**) and axial T1-weighted fat-saturated post-contrast (**B**) images, a new biopsy was planned, targeting the lesion’s central portion (yellow circle), which demonstrated the most conspicuous signal abnormalities and post-contrast enhancement, to improve diagnostic yield. Histopathologic analysis confirmed the diagnosis of primary bone lymphoma. A trans iliac approach was chosen to access this region safely (**C**, **D**), avoiding the sacral neuroforamina and nerve roots. When using trans iliac or other long intraosseous trajectories, precise needle alignment at the outset is critical, as once the needle penetrates deeper into the bone, trajectory adjustments become unfeasible

**Fig. 5:**
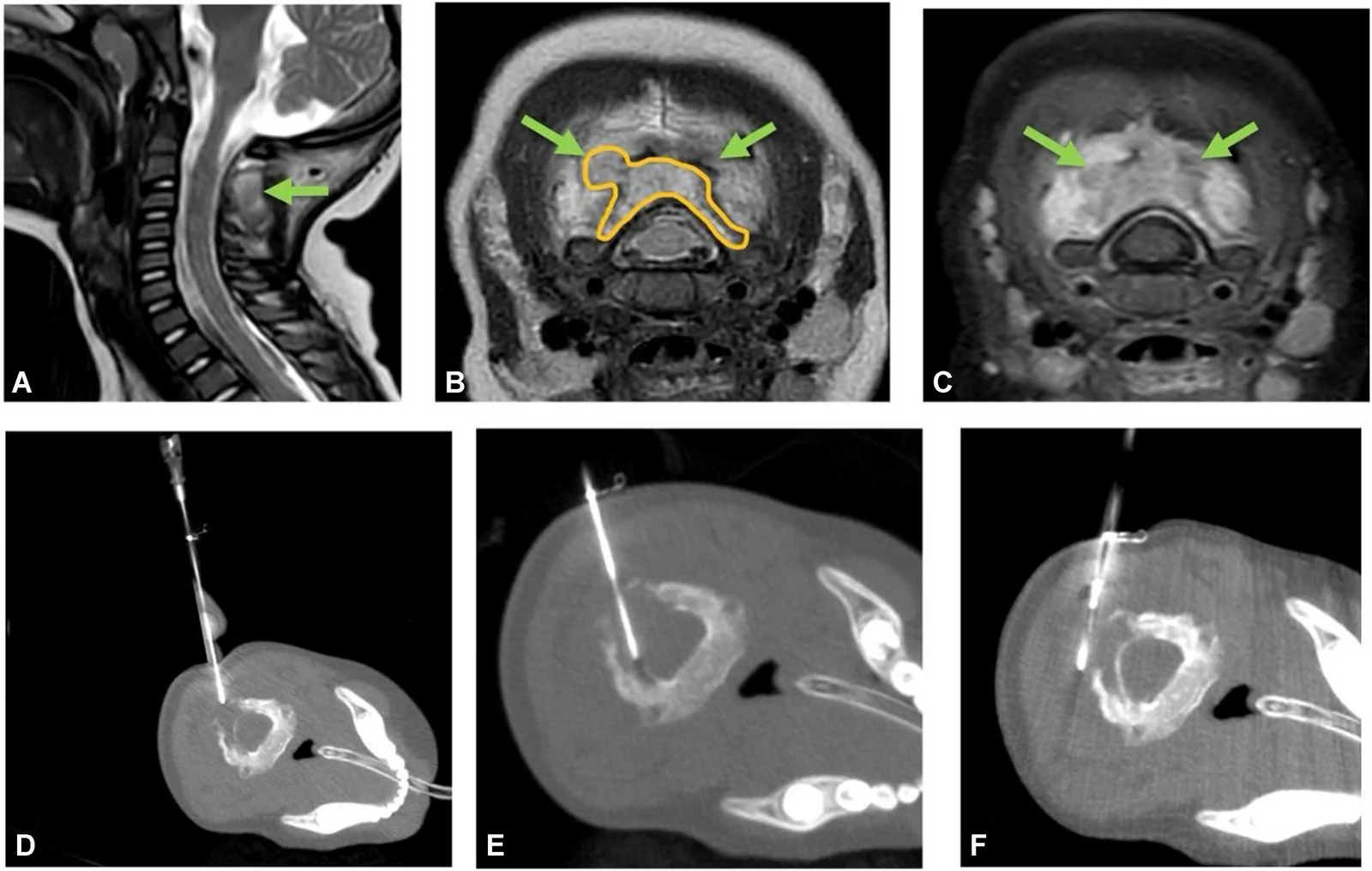
A 3-year-and-7-month-old boy presented with restricted neck mobility following trauma. Sagittal T2-weighted (**A**), axial T2-weighted (**B**), and axial T1-weighted fat-saturated post-contrast (**C**) MR images demonstrate a lesion involving the posterior arch of C2 (arrows), with contrast enhancement and surrounding soft tissue edema. The lesion, outlined in yellow, shows a focus of extraosseous extension. Given the marked cortical thinning and osteolytic nature of the lesion, a soft-tissue biopsy needle was selected for sampling. The biopsy was performed via a posterolateral approach using a 17G coaxial needle and an 18G core biopsy needle. After coaxial needle placement adjacent to the lesion (**D**), a soft-tissue biopsy needle was advanced and carefully positioned to sample both intraosseous and extraosseous components (**E**, **F**), under real-time CT fluoroscopic guidance. A shorter cutting length was used to reduce the risk of inadvertent penetration into the spinal canal. Final histopathologic diagnosis: eosinophilic granuloma. This case illustrates the critical role of meticulous anatomical planning in pediatric spine biopsies, given the narrow pedicles and small epidural spaces surrounding neural structures

Similarly, sacral and pelvic lesions situated close to sacral foramina, the lumbosacral plexus, or iliac vessels often require long transosseous trajectories, such as trans iliac or trans-sacroiliac access, to avoid neurovascular structures and to allow stable anchorage within the bone (Fig. 4) [7, 8, 12, 13, 18, 19, 25]. Long intraosseous paths demand meticulous control of needle orientation in multiple planes and may benefit from CT fluoroscopy (Fig. 5), cone-beam CT, or navigation systems to maintain precision while minimizing cumulative radiation dose and procedure time [1, 4, 7, 8, 18, 19, 25].

**Fig. 6:**
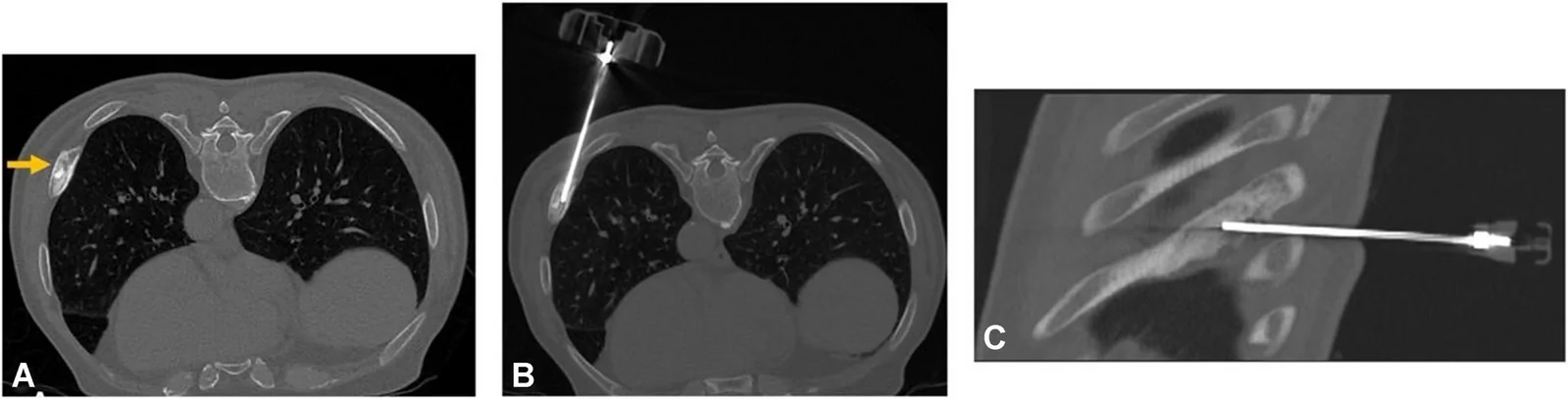
A 75-year-old man with a known diagnosis of bladder carcinoma presented with a new mixed lytic and sclerotic lesion with subtle ground-glass matrix in the left posterior rib, suspicious for metastasis (**A**). In this case, a trajectory parallel to the rib was selected to minimize the risk of pneumothorax and lung injury (**B**). Histopathologic evaluation revealed fibrous dysplasia. On sagittal plane reconstruction (**C**), the entry angle into the rib was suboptimal due to the natural curvature and inclination of the bone. This anatomical feature increases the risk of needle slippage and inadvertent damage to surrounding structures. To mitigate this, special care was taken to stabilize the needle with both hands and to apply slight cranial pressure during initial bone entry. This case illustrates a key principle for rib biopsies: maximizing parallelism to the bone surface to ensure safe access and reduce the potential for complications

**Fig. 7:**
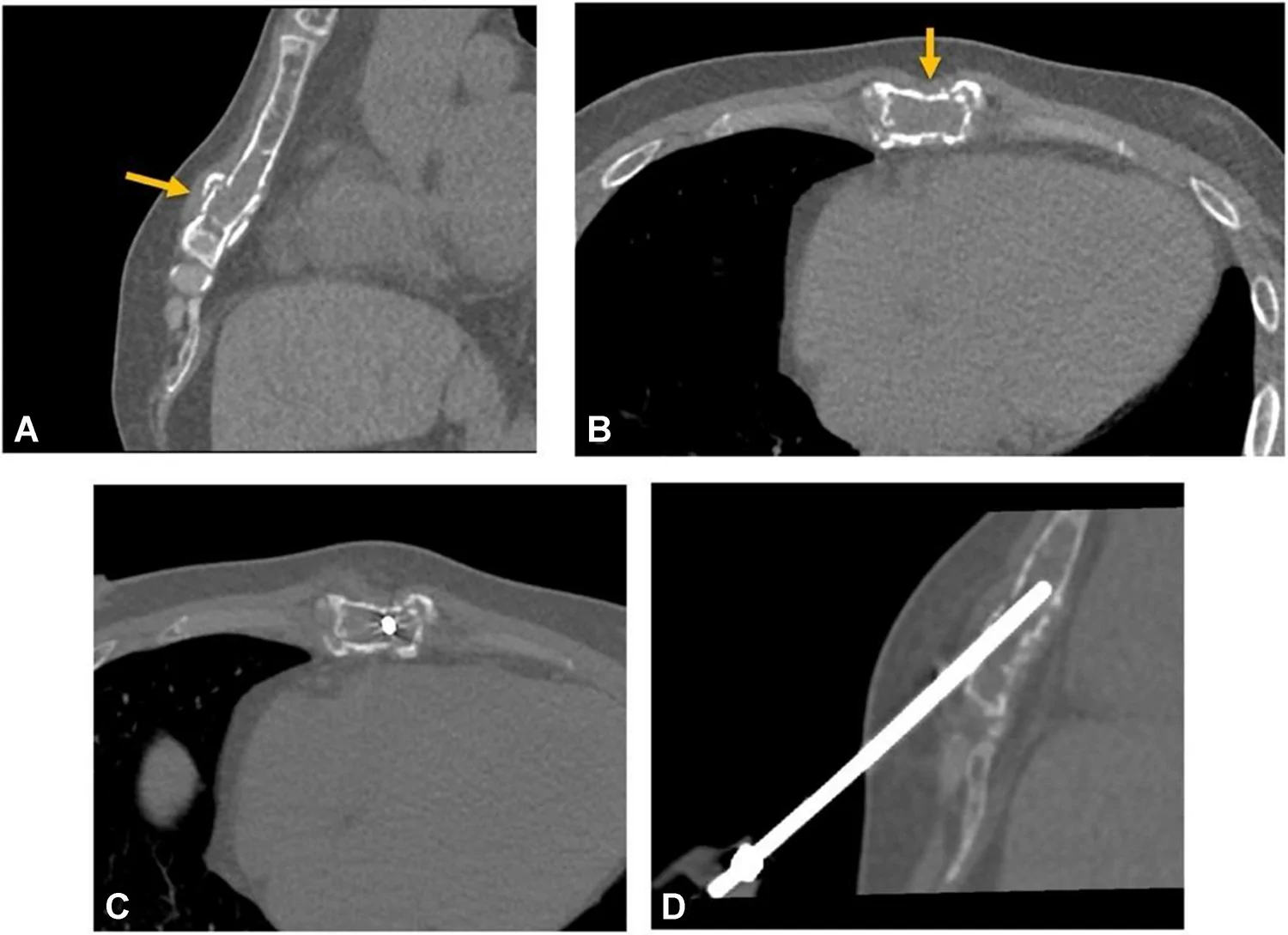
A 59-year-old man presented with an aggressive osteolytic lesion involving the sternum (**A**, **B**). A trajectory as parallel as possible to the cortical surface was preferred to minimize the risk of transfixion and injury to adjacent structures such as the lungs or heart (**C**, **D**). In this case, the biopsy was performed along the long axis of the lesion to maximize core length and improve diagnostic yield. Histopathologic analysis confirmed the diagnosis of plasmacytoma. This case highlights the need for carefully tailored trajectories in sternal biopsies to respect bone thickness and mediastinal anatomy

Lesions in the sternum, ribs, and upper thoracic spine carry a risk of pleural or mediastinal violation [1, 4, 7, 8, 18, 19, 25]. For rib lesions, a near-parallel trajectory maximizes core length and reduces pneumothorax risk; because of the curved cortex, a more perpendicular initial angle may be needed before flattening the needle along the rib (Fig. 6). For sternal and manubrial targets, a shallow anterior approach with frequent CT checks helps respect bone thickness and mediastinal anatomy (Fig. 7). Multiplanar review and alternative oblique intraosseous routes should be considered when standard access is unsafe [1, 3, 4, 6, 7, 10, 12, 18, 19, 21, 22].

### CT-occult lesions and the role of PET/CT and MRI

A third challenging group comprises lesions that are occult or poorly conspicuous on unenhanced CT, but clearly evident on MRI, bone scintigraphy, or PET/CT. These include early marrow metastases, hematologic malignancies, treated or residual tumor foci, and certain infectious lesions whose conspicuity relies more on marrow signal or metabolic activity than on mineralized matrix change. Robust data specifically reporting diagnostic yield for bone lesions occult on CT are limited, as these cases are typically included within broader biopsy series without dedicated subgroup analysis [1, 4, 5, 14, 18, 34–38].

**Fig. 8:**
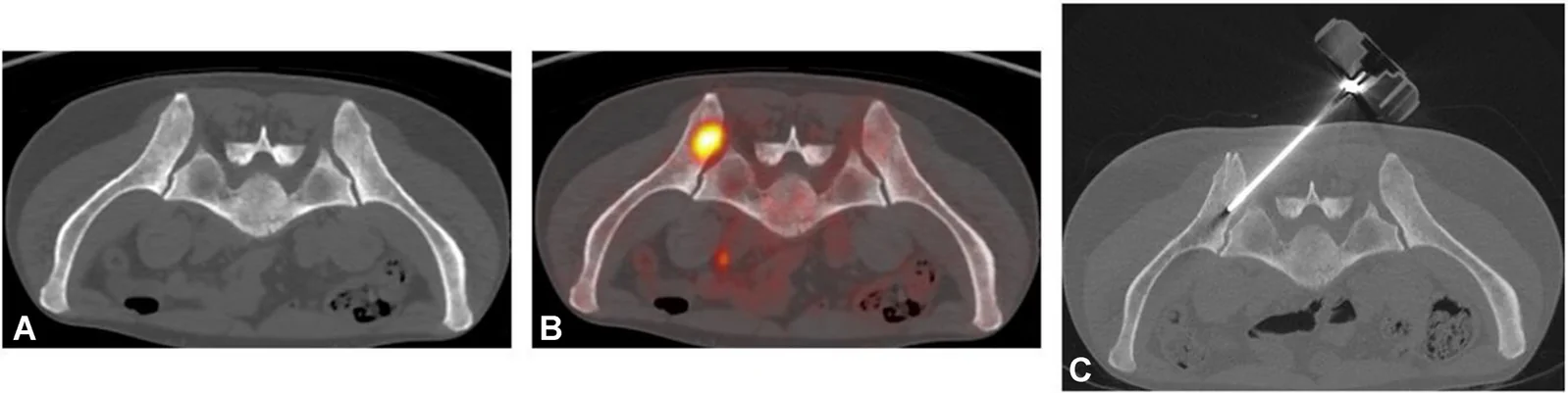
CT-occult pelvic lesion targeted with PET/CT-guided biopsy. A 36-year-old man with a history of diffuse large B-cell non-Hodgkin lymphoma demonstrated a hypermetabolic focus in the left iliac bone on 18F-FDG PET/CT images (**B**), without a corresponding abnormality on standard CT images (**A**). PET/CT was used to localize the most metabolically active portion of the bone marrow and to guide needle placement. Under CT guidance, the biopsy needle was advanced to the PET-avid region, and core samples were obtained (**C**). Histopathologic analysis confirmed recurrent B-cell lymphoma. This case exemplifies the role of PET/CT in guiding biopsy of CT-occult marrow lesions and in improving diagnostic yield when CT findings are subtle or inconspicuous

**Fig. 9:**
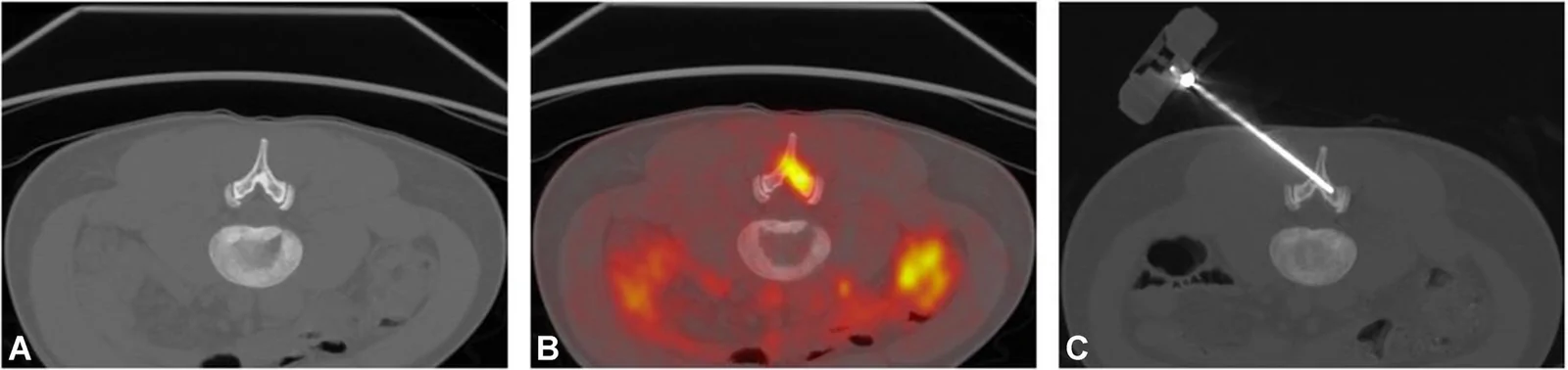
PET-positive vertebral lesion with negative CT-guided biopsy in a patient with Hodgkin lymphoma. A 27-year-old man with Hodgkin lymphoma underwent post treatment 18F-FDG PET/CT, which demonstrated a new hypermetabolic focus in the posterior elements of L3 (**B**), suspicious for residual or recurrent disease, without a corresponding abnormality on standard CT images (**A**). CT-guided biopsy was performed (**C**), with needle placement planned using anatomical landmarks to ensure accurate targeting of the PET-positive site. Histopathologic and microbiologic analyses were negative for malignancy or infection. Subsequent follow-up imaging and clinical assessment showed complete resolution of the PET abnormality, with no structural lesion identified on MRI or CT. This case illustrates that PET-positive osseous foci may occasionally represent false-positive findings, underscoring the importance of image guided biopsy and multimodality correlation before major changes in oncologic management
- Use of interventional MRI units with real-time MR guidance**,** which permits direct visualization of marrow signal abnormalities during needle advancement, particularly useful in small or purely intramedullary lesions [1, 4, 18].
- Real-time PET/CT guidance with robotic arm–assisted needle navigation or PET/CT-guided percutaneous biopsy**,** especially for hypermetabolic bone lesions, which has been associated with very high diagnostic yields and a significant impact on clinical decision-making [1, 5, 18, 34, 35].
- Dual-energy CT with bone marrow settings and virtual non-calcium reconstructions**,** which can unmask otherwise subtle marrow abnormalities that are invisible on standard monoenergetic CT, thereby creating a CT-visible target suitable for conventional guidance [4, 18, 36–38].

When PET- or MRI-positive lesions lack a CT correlate, conventional CT guidance may not confidently localize the highest-yield target. Complementary strategies include the following:

- Anatomic-landmark–based targeting translates the lesion from MRI or PET/CT to CT using fixed osseous landmarks such as cortical contours, joint spaces, endplates, or pedicles. This method can achieve good diagnostic yield (Figs. 8 and 9) [4]. Positional differences between diagnostic imaging and biopsy should be considered, particularly in the pelvis and spine.

Across all approaches, target the most metabolically active, enhancing, or progressive viable tissue rather than necrotic centers or heavily sclerotic rims [1, 4, 7–9, 12, 16, 18, 19, 22]. For small lesions, multiple slightly redirected passes can be obtained through a single cortical entry.

### Complications of CT-guided bone biopsy

Complications of CT-guided bone biopsy are uncommon, with overall rates below 5% in most series [7, 10–13, 16, 18, 19, 22, 25, 26, 39, 40]. Nevertheless, the risk profile may be slightly higher in unusual or technically demanding scenarios, and radiologists should be familiar with both the expected spectrum of events and practical management strategies.

Reported complications include bleeding, infection, medication or contrast reactions, injury to adjacent structures, postprocedural pain, pathologic fracture, and needle bending or fracture [7, 10, 12–14, 18, 19, 25, 39, 40]. Fracture is rare but deserves attention in structurally weakened, weight-bearing bones such as the femoral neck. Postprocedural instructions should reflect lesion location, biopsy size, and cortical integrity and may include temporary activity modification or protected weight bearing [7, 10, 12, 13, 15, 17, 18, 25, 41]. Near joints or hardware, contamination or iatrogenic damage should be weighed against diagnostic benefit [3, 6, 10, 12, 18, 19, 22].

In cases of excessive bleeding along the biopsy tract, several measures can be taken. Manual compression and careful postprocedural observation are often sufficient; however, if bleeding persists, an absorbable gelatin sponge (e.g., Gelfoam) can be fragmented, mixed with saline, and injected as a slurry through the outer working cannula at the end of the procedure to promote tract hemostasis [7, 8, 12, 16, 18]. This technique is particularly useful in hypervascular tumors of the pelvis and spine or in patients receiving anticoagulant or antiangiogenic therapies. Only in exceptional situations is endovascular embolization or surgical intervention required [7, 10, 12, 13, 18, 25, 39, 40].

**Fig. 10:**
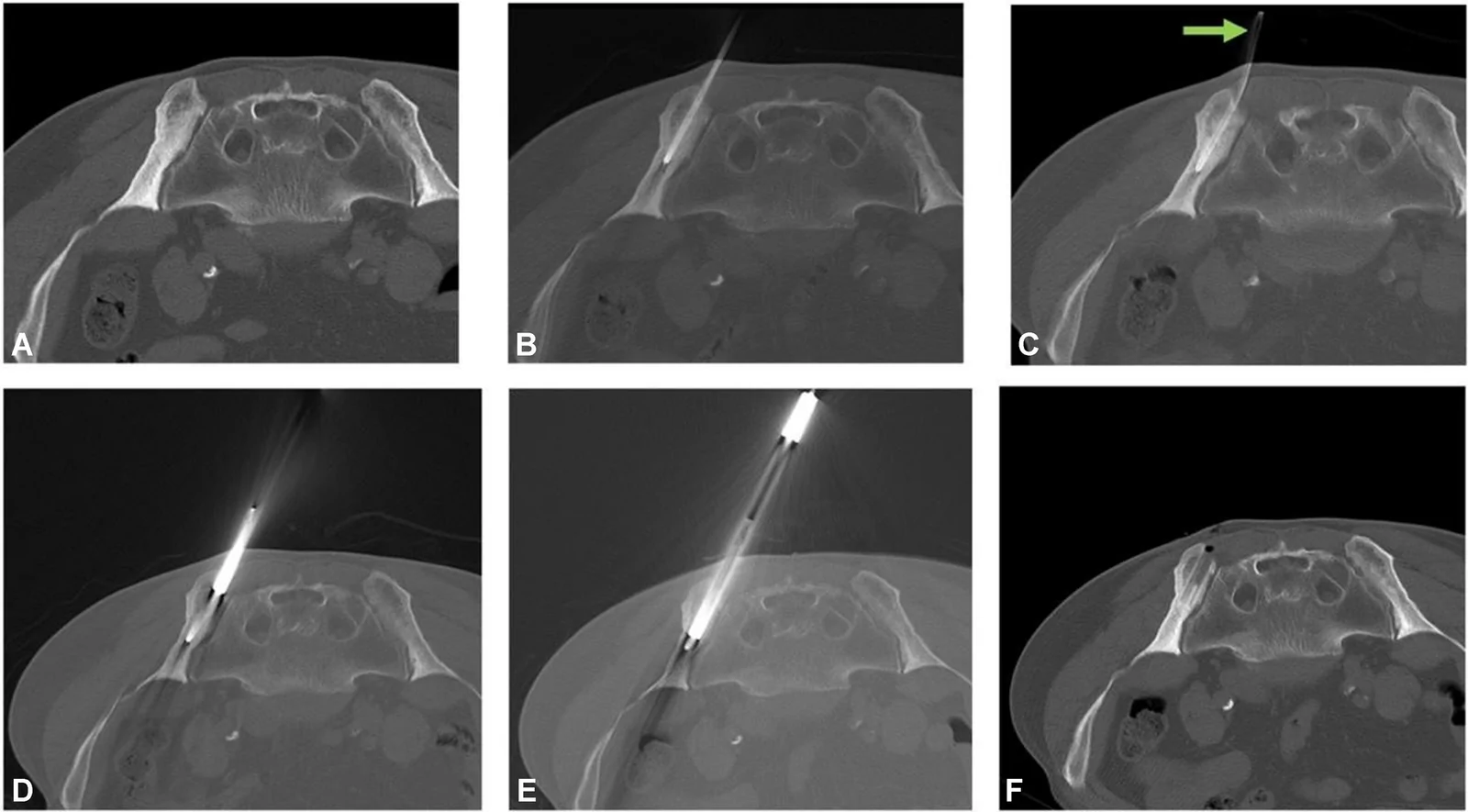
Needle fracture during CT-guided biopsy of a densely sclerotic iliac bone lesion. A 65-year-old man presented with nonspecific areas of sclerosis in the iliac bones (**A**), and a biopsy was requested to exclude malignancy. This case illustrates a procedural complication, in which the biopsy needle became deeply embedded in the sclerotic lesion (**B**), resulting in fracture of the needle base during manipulation (**C**, arrow). This scenario underscores the technical challenges and potential risks associated with biopsy of dense, sclerotic bone. Retrieval of the retained needle fragment was performed in collaboration with the orthopedic oncology team, using a larger-gauge trephine (**D**, **E**) to encompass the broken needle along with surrounding bone (**F**), which was then submitted for histopathological evaluation. No malignancy was identified

Needle fracture or broken hardware is a rare but characteristic complication, especially in densely sclerotic lesions when aggressive torque is applied or the cortex is traversed at an unfavorable angle (Fig. 10) [7, 8, 12, 15, 17, 18, 25, 26]. Completely intraosseous retained fragments can often be safely left in place if they do not cross a joint or interfere with planned surgical instrumentation; otherwise, percutaneous retrieval using snares, forceps, or needle-in-needle techniques should be attempted [7, 8, 12, 15, 17, 18, 25, 26].

Non-diagnostic sampling should be minimized through representative targeting, sufficient cores, and coordination with pathology. When available, intraprocedural frozen section may confirm adequacy and reduce repeat biopsy [7–9, 11, 12, 16–19, 29].

## Conclusion

CT-guided percutaneous bone biopsy is a reliable, minimally invasive diagnostic tool. In challenging lesions, success depends on careful target and trajectory selection, appropriate device and sampling strategy, and awareness of complications. Tailoring technique to lesion density, conspicuity, and adjacent anatomy helps maximize diagnostic yield and safety.

## Data Availability

Data can be shared on request.
